# Successful lung transplantation in RPILD associated with anti-MDA5ab+: A case report

**DOI:** 10.1097/MD.0000000000041408

**Published:** 2025-02-14

**Authors:** Chaoyang Zhang, Jianxing Guo, Guowei Ye, Dong Zhang

**Affiliations:** a Department of Intensive Care Unit, The First Hospital of Jilin University, Changchun, Jilin, China.

**Keywords:** antimelanoma differentiation–associated gene 5 antibody, extracorporeal membrane oxygenation, lung transplantation, RPILD

## Abstract

**Rationale:**

Lung transplantation in rapidly progressive interstitial lung disease associated with antimelanoma differentiation–associated gene 5 antibody positive may be a rescue therapy in case of medical treatment failure and impossible weaning from the ventilatory support.

**Patient concerns:**

A 59-year-old man with cough, expectoration, and fever for more than half a month, which aggravated with dyspnea for 4 days.

**Diagnoses:**

Rapidly progressive interstitial lung disease was confirmed through chest high-resolution computed tomography and antimelanoma differentiation–associated gene 5 antibody positive.

**Interventions:**

The patient was given extracorporeal membrane oxygenation and double lung transplantation.

**Outcomes:**

The patient is currently undergoing rehabilitation in a general ward and follow-up.

**Lessons:**

Lung transplantation should be considered when impossible weaning from the ventilatory support.

## 1. Introduction

According to the 2019 International Society for Heart and Lung Transplantation Registry report, the proportion of lung transplantation performed in patients with end-stage lung disease related to connective tissue disease is small (0.9%).^[1]^ Dermatomyositis is a group of connective tissue disease characterized by muscular and cutaneous inflammation.^[2]^ The antimelanoma differentiation–associated gene 5 antibody positive (anti-MDA5ab+) is present in 10% to 35% of patients and is frequently associated with clinical amyopathic dermatomyositis and a high risk of rapidly progressive interstitial lung disease (RPILD).^[3]^ The 6-month survival rate in some studies is 40% despite the therapies described.^[4]^ Treatment usually associates glucocorticoids with immunosuppressants/modulators (e.g., cyclophosphamide, mycophenolate mofetil, calcineurin inhibitor, rituximab, or intravenous immunoglobulin).^[5]^ Lung transplantation in RPILD associated with anti-MDA5ab+ may be a rescue therapy in case of medical treatment failure and impossible weaning from the ventilatory support.^[3,6]^

We report the case of the fastest bridge to double lung transplantation related to anti-MDA5ab+ with extracorporeal membrane oxygenation (ECMO).

## 2. Case report

A 59-year-old man with a 6-year history of type 2 diabetes and hypertensive disease was hospitalized on July 2, 2024, due to cough, expectoration, and fever for more than half a month, which aggravated with dyspnea for 4 days. The patient was treated with oxygen therapy, methylprednisolone, meropenem, nebulization, and symptomatic treatment after admission.

**Figure 1.**
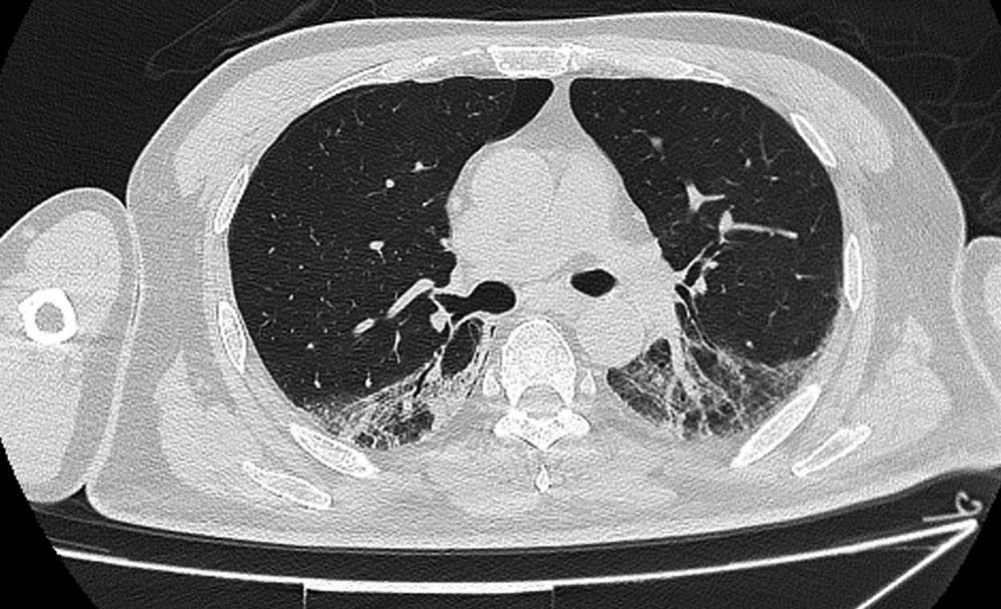
Chest high-resolution computed tomography on July 7, 2024 showed a few ground glass shadows in both lungs.

On July 4, 2024, the 24-item myositis spectrum test results for the patient showed a positive reaction for anti-MDA5 antibodies with a titer of 39 AU. In conjunction with the patient’s chest high-resolution computed tomography findings (Fig. 1), this suggests a diagnosis of RPILD with anti-MDA5ab+. Our diagnosis was confirmed by the pathological diagnosis report. Pathological diagnoses are reported in Supplementary File S1, Supplemental Digital Content, http://links.lww.com/MD/O328. The patient was treated with first-line and second-line immunotherapies including methylprednisolone, 2 rounds of human immunoglobulin, tacrolimus, tocilizumab, and tofacitinib, as well as anti-infection, antiviral, and antifungal treatments.

**Figure 2.**
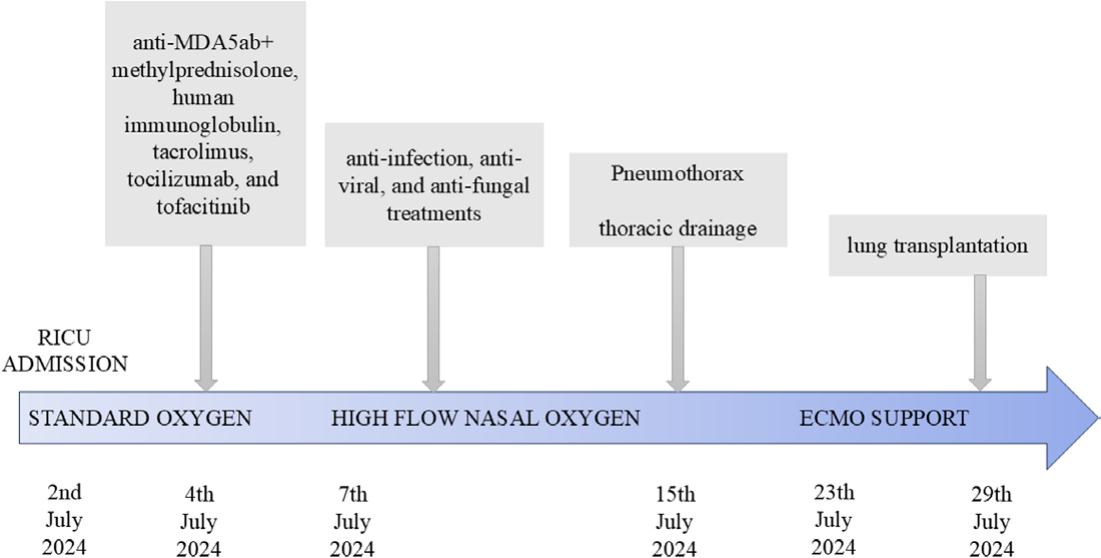
The timeline summarizes the successive treatments administered to the patient after diagnosis of rapidly progressive interstitial lung disease. Anti-MDA5ab+ = antimelanoma differentiation–associated gene 5 antibody positive, ECMO = extracorporeal membrane oxygenation.

A right pneumothorax occurred after the treatment. The lung expansion was poor, and the oxygenation index progressively decreased after thoracic drainage. On July 23, 2024, adequate oxygenation was not achieved (PaO_2_/FiO_2_ ratio < 75) despite maximal ventilatory support, leading us to start v-v ECMO (right femoral/ right jugular veins) as a bridge to lung transplantation (Fig. 2). Two days later, the results of muscle enzymes were negative for anti-MDA5ab, with a titer <5 AU. However, it was irreversible for the patient’s respiratory damage. After evaluation by the thoracic surgery department, the patient underwent an emergency double lung transplantation under general anesthesia after 6 days of bridging v-v ECMO. The donor was a previously healthy patient with brain death due to intracerebral hemorrhage with no known history of lung disease.

The patient was transferred to the intensive care unit after surgery and was weaned off the ventilator and extubated on postoperative day 6, with sequential respiratory support using a noninvasive ventilator and high-flow nasal cannula. The patient developed bloodstream infection, sepsis, and acute kidney injury after operation. Early postoperative care focuses on ventilatory support and weaning, fluid and hemodynamic management, immunosuppression, detection of early rejection, and prevention or treatment of infection. Infection has been one of the leading causes of early morbidity and mortality. In the perioperative period, bacterial pathogens are the greatest threat. Prophylaxis against bacterial infection is routinely administered perioperatively. In the absence of specific culture results, we use an initial empiric regimen, which expanded to include coverage of potential pathogens that have been isolated from the donor or recipient, as well as local infectious considerations. Postoperatively, we have implemented a triple-drug maintenance immunosuppression regimen, which includes tacrolimus, corticosteroids, and mycophenolate mofetil. After active anti-infection, hemofiltration, and antirejection symptomatic and supportive treatment, the patient was transferred to the general rehabilitation ward for further rehabilitation treatment. Following a comprehensive 15-day rehabilitation program, the patient’s vital signs became stable, and upon meeting the discharge criteria, the patient was discharged from the hospital. After 3 months, the patient returned to the hospital for a follow-up visit, during which it was confirmed that the anti-MDA5 antibodies were negative. The patient has since resumed a normal life.

## 3. Discussion

In individuals afflicted with RPILD, especially those who test positive for antimelanoma differentiation-associated gene 5 antibodies, the criteria and timing for lung transplantation are of paramount importance in the clinical decision-making process. Lung transplantation surfaces as a potential life-saving intervention when all pharmacological options have been exhausted and patients find themselves incapable of discontinuing respiratory assistance. A study conducted by Leclair et al^[6]^ in the year 2018 adeptly illustrated the implementation of lung transplantation in patients afflicted with RPILD who are differentiation-associated gene 5 antibodies positive, underscoring the critical nature of considering lung transplantation as a therapeutic alternative when traditional therapeutic approaches prove to be futile.

The assessment of postoperative prognosis and survival rates is integral to evaluating the success of lung transplantation as a therapeutic intervention. A retrospective cohort study conducted by Ye et al,^[4]^ focusing on adult patients with clinically amyopathic dermatomyositis associated with RPILD, revealed that lung transplantation can markedly enhance patient survival rates. This study offers persuasive evidence supporting the potential therapeutic advantages of lung transplantation for individuals battling RPILD, thereby reinforcing its consideration as a valuable treatment option in such clinical scenarios.

The oversight of postoperative complications following lung transplantation is paramount for guaranteeing both long-term survival and the quality of life for patients. In a study conducted by Bay et al,^[7]^ which focused on individuals with RPILD positive for antimelanoma differentiation-associated gene 5 antibodies who underwent lung transplantation with the aid of extracorporeal life support, the critical nature of infection control, and the prompt identification of rejection episodes were underscored. These strategic management approaches are fundamental in enhancing patient survival rates and optimizing overall outcomes posttransplantation.

Weighing the efficacy and risks of lung transplantation against alternative therapeutic approaches, such as immunosuppressive therapy, in the context of RPILD presents a multifaceted challenge. A multicenter, prospective study led by Tsuji et al^[5]^ offered a comparative analysis between the impact of a comprehensive immunosuppressive regimen, which included high-dose glucocorticoid, tacrolimus, and cyclophosphamide, and the outcomes of lung transplantation. This study provided valuable insights and reference points for clinicians when making critical decisions regarding the management of RPILD. The findings contribute to the body of knowledge that aids in discerning between the potential benefits and risks associated with each treatment option, thereby informing personalized treatment plans for patients with RPILD.

The ethical considerations encircling lung transplantation are multilayered and require the collective attention of both the medical community and society at large. These include, but are not limited to, the principles of fairness and the criteria for patient selection in the process of organ allocation. The International Thoracic Organ Transplant Registry’s report, authored by Chambers et al,^[1]^ delved into critical topics such as the matching of donor and recipient sizes. This discussion is central to ensuring that the distribution of organs is not only equitable but also conducted with the highest degree of efficiency. The report underscores the necessity for transparent and just protocols in organ allocation to address the complex interplay between medical necessity, ethical imperatives, and societal values.

Investigative efforts moving forward must be dedicated to uncovering innovative treatment methodologies and enhancing the technical aspects of lung transplantation. A retrospective analysis conducted by Fan et al,^[8]^ which examined the outcomes of patients with melanoma differentiation-associated gene 5-related interstitial lung disease treated with either tofacitinib or tacrolimus, has yielded valuable data. This analysis has not only shed light on the comparative efficacy of these treatments but also provided a compass for the evolution of future therapeutic strategies. Such research is instrumental in guiding the medical community toward more effective care pathways for patients afflicted with melanoma differentiation-associated gene 5-related interstitial lung disease, potentially leading to advancements that may improve patient outcomes in the context of lung transplantation and beyond.

Given the few case reports of lung transplantation in this context, this procedure seems to be underused. For our patient, this rescue therapy was successful despite multiple postoperative complications. The China Organ Transplant Response System automatically allocates organs based on factors such as the severity of the patient’s condition, waiting time, and geographical location, allowing us to perform transplantation 6 days after the beginning of ECMO support, faster than reported in other series.^[3,9–13]^ There is no doubt that highly selected patients can benefit from this technique.

## 4. Conclusions

This report confirms the interest of lung transplantation in RPILD related to anti-MDA5ab+. Long-term results are not available in the literature, and the risk of relapse is unknown, but longer follow-up could answer this question. New treatment strategies are needed to avoid this ultimate life-saving intervention, particularly in an emergency context. In the meantime, intensivists must be aware of the possibility of lung transplantation in this context and refer patients to specialized centers when they are young and have no comorbidities.

## Author contributions

**Writing – original draft:** Chaoyang Zhang.

**Investigation:** Jianxing Guo, Guowei Ye.

**Writing – review & editing:** Dong Zhang.

## Supplementary Material


