# Supplementary material for: Successful lung transplantation in RPILD associated with anti-MDA5ab+: A case report
Source: Medicine (Baltimore). 2025 Feb 14;104(7):e41408. doi: 10.1097/MD.0000000000041408 (PMC11835130; doi:10.1097/MD.0000000000041408)
Supplement: Supplementary file 1 [file medi-104-e41408-s001.pdf]

吉林大学白求恩第一医院

病理诊断报告

病理号：1059230A　　申请科室：胸外二科

姓名：白瑜　　性别：男　　年龄：59岁
住院号：12742198　　病区：呼吸科RICU护理组　　送检医师：李洋
门诊号：　　ID号：1352097493　　接收日期：2024年07月30日

临床诊断：重症肺炎

标本类型及肉眼所见：

1.（左全肺）送检左侧全肺，总重356g，上叶体积10.5cm*10cm*4cm，下叶体积10.5cm*8cm*7cm，切面淡黄及淡褐色、偏实性、质略韧，距支气管切缘7cm，肺尖处见囊性膨出数枚，最大径0.5cm-2.5cm。支气管旁淋巴结14枚，直径0.5cm-2cm。

2.（右肺）送检肺叶，总重547g，上中叶发育畸形，水平裂模糊，上中叶总体积15cm*11cm*5cm，下叶体积11cm*10cm*5.5cm，距支气管切缘7cm，中叶见囊性膨出数枚，最大径0.5cm-2cm。距支气管切缘8cm，下叶见囊性膨出数枚，直径0.3cm-1.5cm。支气管旁淋巴结11枚，直径0.5cm-1.3cm。

光镜所见（附图）：

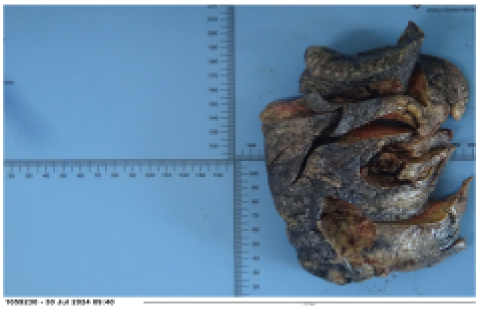

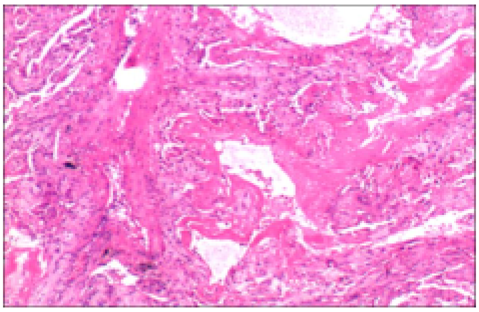

病理诊断：

【左全肺、右肺】胸膜下多个肺小叶肺泡结构消失，纤维组织增生，慢性炎细胞浸润；余肺肺泡间隔增宽，纤维母细胞增生，较多慢性炎细胞浸润，肺泡上皮细胞增生，左肺上叶部分区域可见透明膜形成，部分细支气管粘膜坏死脱落，细支气管腔内及肺泡腔内见纤维素性渗出，伴机化，可见细支气管周化生，部分肺泡腔融合、扩张，大泡形成；支气管旁淋巴结反应性增生（左侧14枚，右侧11枚）；形态符合非特异性间质性肺炎样改变（NSIP样改变），合并弥漫性肺泡损伤（DAD）。

免疫组化：CK7(+),TTF-1(8G7G3/1)(+),CD34(血管+),CD68(+),CD163(+),CD3(少许+),CD20(少许+),CD138(少许+)
特殊染色：Masson(+),弹力纤维(+),网染(+)

会诊医师：马小波　主诊医师：[illegible]　初诊医师：[illegible]　首次报告日期：2024年08月05日

※ 上述诊断如与临床诊断不符，请速与主诊医师联系，电话：0431-81875902。
